# Supplementary material for: Effect of Dietary Supplementation of Lactobacillus Casei YYL3 and L. Plantarum YYL5 on Growth, Immune Response and Intestinal Microbiota in Channel Catfish
Source: Animals (Basel). 2019 Nov 20;9(12):1005. doi: 10.3390/ani9121005 (PMC6941169; doi:10.3390/ani9121005)
Supplement: Supplementary file 1 [file animals-09-01005-s001.pdf]

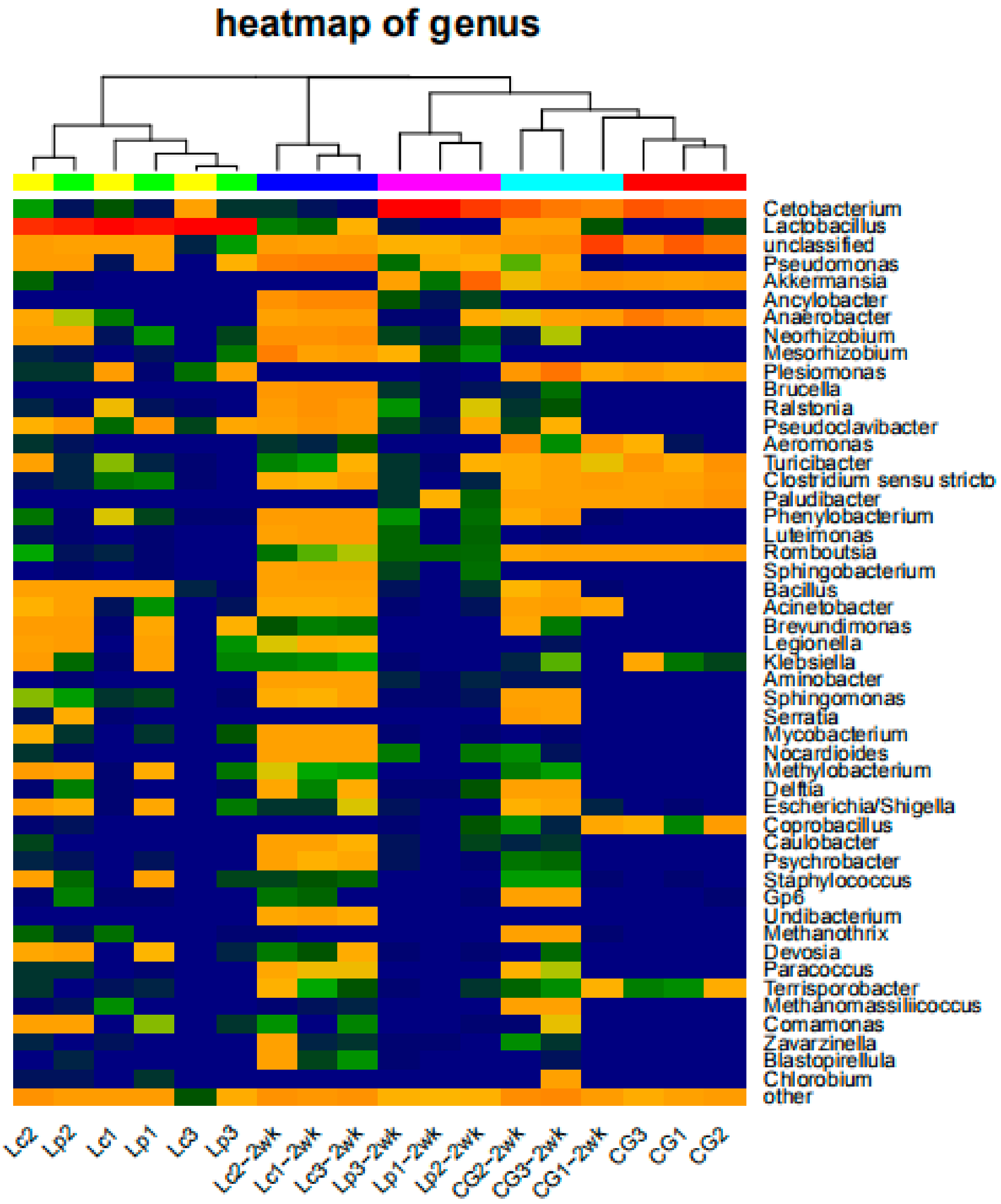

Fig.S1. Heatmap showing the gut microbial compositions of channel catfish right after feeding with basal diet and *Lactobacillus*-containing diets for 4 weeks and after 2 more weeks cessation.The heatmap was generated based on the SIMPER (similarity percentages procedure) result, which represents percentage contribution of each taxon from the channel catfish samples. The SIMPER analysis and heatmap visualization were performed using the vegan package of R statistical software. (CG1, CG2 and CG3: 3 samples from control group; Lc1, Lc2 and Lc3: 3 samples from Lc-diet group; Lp1, Lp2 and Lp3: 3

samples from Lp-diet group; CG1-2wk, CG2-2wk and CG3-2wk: 3 samples from control group that are fed with basal diet for two more weeks; Lc1-2wk, Lc2-2wk, Lc3-2wk: 3 samples from Lc group that are fed with basal diet for two more weeks; Lp1-2wk, Lp2-2wk, Lp3-2wk: 3 samples from Lp group that are fed with basal diet for two more weeks.

Table S1. The first 10 genera in the 6 groups at genus level

Abundance rank	Genus subdivision by source					
	CG	Lc	Lp	CG-2wk	Lc-2wk	Lp-2wk
1	*Cetobacterium*	*Lactobacillus*	*Lactobacillus*	*Cetobacterium*	*Pseudomonas*	*Cetobacterium*
2	*unclassified*	*Pseudomonas*	*Pseudoclavibacter*	*unclassified*	*Ancylobacter*	*Akkermansia*
3	*Anaerobacter*	*unclassified*	*Pseudomonas*	*Plesiomonas*	*Neorhizobium*	*unclassified*
4	*Turicibacter*	*Klebsiella*	*Legionella*	*Aeromonas*	*Mesorhizobium*	*Pseudomonas*
5	*Paludibacter*	*Brevundimonas*	*Brevundimonas*	*Akkermansia*	*Brucella*	*Pseudoclavibacter*
6	*Clostridium sensu stricto*	*Plesiomonas*	*unclassified*	*Clostridium sensu stricto*	*Ralstonia*	*Mesorhizobium*
7	*Akkermansia*	*Methylobacterium*	*Bacillus*	*Serratia*	*Pseudoclavibacter*	*Anaerobacter*
8	*Romboutsia*	*Bacillus*	*Methylobacterium*	*Acinetobacter*	*Phenylobacterium*	*Turicibacter*
9	*Plesiomonas*	*Cetobacterium*	*Klebsiella*	*Anaerobacter*	*unclassified*	*Ralstonia*
10	*Coprobacillus*	*Escherichia/Shigella*	*Escherichia/Shigella*	*Phenylobacterium*	*Luteimonas*	*Paludibacter*
11	*Anaerofilum*	*Turicibacter*	*Devosia*	*Paludibacter*	*Anaerobacter*	*Phenylobacterium*

Table S2. Mean relative abundances (%) of selected KEGG pathways related to metabolism.

KEGG level	Pathway	Abundance (%)		
		CG	Lc	Lp
**1**	**Metabolism**	2.765	3.795	3.215
**2**	**Energy Metabolism**	5.869	4.931^{a*}	4.844$^{b\#}$
3	Carbon fixation pathways in prokaryotes	1.252	0.968^{a*}	0.893$^{b\#}$
3	Nitrogen metabolism	0.844	0.577^{a*}	0.604$^{b\#}$
3	Carbon fixation in photosynthetic organisms	0.625	0.485^{a*}	0.504$^{b\#}$
**2**	**Lipid Metabolism**	2.964	3.491^{a*}	3.374
3	Fatty acid elongation in mitochondria	0.002	0.000^{a*}	0.000$^{b\#}$
3	Glycerophospholipid metabolism	0.581	0.518^{a*}	0.518
3	Fatty acid biosynthesis	0.456	0.804^{a*}	0.706
3	Primary bile acid biosynthesis	0.019	0.099^{a*}	0.072
3	Ether lipid metabolism	0.011	0.002^{a*}	0.003$^{b\#}$
3	Steroid hormone biosynthesis	0.037	0.006^{a*}	0.005$^{b\#}$
3	Secondary bile acid biosynthesis	0.019	0.096^{a*}	0.069
3	Steroid biosynthesis	0.006	0.0022	0.0015$^{b\#}$
**2**	**Amino Acid Metabolism**	10.094	8.750^{a*}	8.900$^{b\#}$
3	Cysteine and methionine metabolism	1.070	0.868^{a*}	0.828$^{b\#}$
3	Glycine, serine and threonine metabolism	0.916	0.712^{a*}	0.750
3	Tyrosine metabolism	0.439	0.667^{a*}	0.585
3	Phenylalanine, tyrosine and tryptophan biosynthesis	0.819	0.714^{a*}	0.657
**2**	**Metabolism of Other Amino Acids**	1.649	1.842^{a*}	1.808
3	D-Glutamine and D-glutamate metabolism	0.173	0.120^{a*}	0.113$^{b\#}$
3	Valine, leucine and isoleucine biosynthesis	0.723	0.375^{a*}	0.433$^{b\#}$
3	Arginine and proline metabolism	1.245	0.835^{a*}	0.910$^{b\#}$
3	Glutathione metabolism	0.266	0.386^{a*}	0.417$^{b\#}$
3	Cyanoamino acid metabolism	0.248	0.174^{a*}	0.200
3	D-Alanine metabolism	0.097	0.176	0.168
**2**	**Metabolism of Cofactors and Vitamins**	4.445	3.346^{a*}	3.314$^{b\#}$
3	Lipoic acid metabolism	0.024	0.077^{a*}	0.075$^{b\#}$
3	Retinol metabolism	0.039	0.085^{a*}	0.074
3	Vitamin B6 metabolism	0.203	0.134^{a*}	0.141$^{b\#}$
3	Nicotinate and nicotinamide metabolism	0.458	0.347^{a*}	0.356$^{b\#}$
3	Pantothenate and CoA biosynthesis	0.617	0.462^{a*}	0.462$^{b\#}$
3	Porphyrin and chlorophyll metabolism	0.943	0.227^{a*}	0.279$^{b\#}$
3	Biotin metabolism	0.189	0.091^{a*}	0.080$^{b\#}$
3	One carbon pool by folate	0.547	0.449^{a*}	0.470$^{b\#}$
3	Riboflavin metabolism	0.263	0.279^{a*}	0.250

**2**	**Metabolism of Terpenoids and Polyketides**	1.715	2.081[a*]	2.009[b#]
3	Biosynthesis of ansamycins	0.102	0.076[a*]	0.064
3	Carotenoid biosynthesis	0.014	0.072[a*]	0.047
3	Polyketide sugar unit biosynthesis	0.236	0.157[a*]	0.166[b#]
3	Limonene and pinene degradation	0.116	0.281[a*]	0.243
3	Biosynthesis of vancomycin group antibiotics	0.067	0.040[a*]	0.040[b#]
3	Tetracycline biosynthesis	0.163	0.344[a*]	0.270
**2**	**Biosynthesis of Other Secondary Metabolites**	1.018	0.692[a*]	0.768[b#]
3	Novobiocin biosynthesis	0.157	0.091[a*]	0.099
3	Caffeine metabolism	0.002	0.000[a*]	0.000[b#]
3	Isoquinoline alkaloid biosynthesis	0.091	0.014[a*]	0.017[b#]
3	Penicillin and cephalosporin biosynthesis	0.034	0.073[a*]	0.067[b#]
3	Flavonoid biosynthesis	0.014	0.003[a*]	0.007
3	Tropane, piperidine and pyridine alkaloid biosynthesis	0.146	0.059[a*]	0.077[b#]
3	Phenylpropanoid biosynthesis	0.124	0.053[a*]	0.078
3	Streptomycin biosynthesis	0.340	0.279[a*]	0.306
3	Novobiocin biosynthesis	0.157	0.091	0.099[b#]
3	Stilbenoid, diarylheptanoid and gingerol biosynthesis	0.012	0.008	0.005[b#]
**2**	**Xenobiotics Biodegradation and Metabolism**	2.068	2.839[a*]	2.262
3	Drug metabolism - other enzymes	0.351	0.210[a*]	0.235[b#]
3	Polycyclic aromatic hydrocarbon degradation	0.089	0.156[a*]	0.152[b#]
3	Toluene degradation	0.119	0.166[a*]	0.149
3	Dioxin degradation	0.051	0.108[a*]	0.095
3	Naphthalene degradation	0.231	0.343[a*]	0.280
3	Nitrotoluene degradation	0.1269	0.024[a*]	0.017[b#]
3	Xylene degradation	0.046	0.105[a*]	0.083
3	Ethylbenzene degradation	0.074	0.177[a*]	0.140
3	Chlorocyclohexane and chlorobenzene degradation	0.018	0.050	0.052[b#]
3	Metabolism of xenobiotics by cytochrome P450	0.043	0.110	0.111[b#]
3	Drug metabolism - cytochrome P450	0.048	0.113	0.115[b#]
**2**	**Glycan Biosynthesis and Metabolism**	2.556	1.903	1.861
3	Lipopolysaccharide biosynthesis	0.423	0.102[a*]	0.100[b#]
3	N-Glycan biosynthesis	0.028	0.008[a*]	0.018
3	Aminobenzoate degradation	0.159	0.319[a*]	0.292[b#]
**2**	**Carbohydrate Metabolism**	10.835	11.928	12.142
3	Citrate cycle (TCA cycle)	0.944	0.65[4a*]	0.694[b#]

3	Propanoate metabolism	0.606	0.895[a*]	0.848[b#]
3	Ascorbate and aldarate metabolism	0.101	0.228[a*]	0.226[b#]
3	C5-Branched dibasic acid metabolism	0.335	0.1319[a*]	0.150[b#]
3	Pentose and glucuronate interconversions	0.463	0.402[a*]	0.484
**2**	**Nucleotide Metabolism**	4.054	4.295[a*]	4.191

Note: a* means the significant difference between Lc and CG (p <0.05). b# means the significant difference between Lp and CG (p <0.05).
